# Profile of Patients with Dementia or Cognitive Impairment Hospitalized with a Proximal Femur Fracture Requiring Surgery

**DOI:** 10.3390/ijerph19052799

**Published:** 2022-02-28

**Authors:** Claudia Casafont, María Josefa González-Garcia, Ana Marañón-Echeverría, José Luis Cobo-Sánchez, María Bravo, Mercè Piazuelo, Adelaida Zabalegui

**Affiliations:** 1 Subdivision of Research and Teaching in Nursing, Hospital Clínic Barcelona, 08036 Barcelona, Spain; casafont@clinic.cat; 2 Care Quality and Information Systems Unit, Hospital Universitario de Navarra, 31008 Pamplona, Spain; mj.gonzalez.garcia@navarra.es; 3 Traumatology Department, Hospital Universitario de Navarra, 31008 Pamplona, Spain; am.maranon.echeverria@navarra.es; 4 Research and Innovation Department, Hospital Universitario Marqués de Valdecilla, 39008 Santander, Spain; joseluis.cobo@scsalud.es; 5 Escuela Universitaria Clínica Mompía, Universidad Católica de Ávila, 39108 Mompía, Spain; 6 Department of Neurology, Hospital Universitario Marqués de Valdecilla, 39008 Pamplona, Spain; mariabravo19@hotmail.com; 7 Institute for Research Marqués de Valdecilla, Universidad de Cantabria, 39011 Santander, Spain; 8 Traumatology Department, Hospital Clínic Barcelona, 08036 Barcelona, Spain; mpiazuel@clinic.cat

**Keywords:** dementia, hip fracture, nursing care, hospitalization, dependency, elderly, cognitive impairment, pain

## Abstract

This study reports the characteristics of patients with dementia or cognitive impairment hospitalized with a proximal femur fracture requiring surgery. Methods: Multicentric descriptive longitudinal study conducted in three traumatology units, representing high-technology public hospitals across Spain. Data collection took place between August 2018 and December 2019 upon admission to hospital, discharge, one month and three months after discharge. Results: Study participants (*n* = 174) were mainly women (81.6%), and the mean age was 90.7± 6.3 years old. Significant statistical differences were noted in the decline of functional capacity at baseline and one month later, and after three months they had still not recovered. Malnutrition increased from baseline to the one-month follow-up. The use of physical restraints increased during hospitalization, especially bilateral bedrails and a belt in the chair/bed. After one month, 15.2% of patients had pressure ulcers. Although pain decreased, it was still present after three months. Conclusion: Hospitalization after hip surgery for elderly people with dementia or cognitive impairment negatively impacted their global health outcomes such as malnutrition and the development of pressure ulcers, falls, functional impairment and the use of physical restraints and pain management challenges. Hospitals should implement policy-makers’ strategic dementia care plans to improve their outcomes.

## 1. Introduction

Around 50 million people live with dementia worldwide, and 10 million cases are diagnosed every year. Dementia has physical, psychological, social and economic impacts as it is one of the major causes of dependency among older people. Because of the aging population, this number is expected to increase to 78 million in 2030 and 139 million in 2050 [1]. People with dementia (PwD) have an increased risk of hip fractures [2,3]. In fact, a study conducted in the Netherlands showed that 30% of patients admitted with a hip fracture had dementia [4]. However, dementia seems to be underdiagnosed. A review estimated that more than 60% of people with dementia are undetected [5].

Commonly, PwD have a decline in functional capacity and reduced muscle strength. Thus, falls are associated with impaired cognition, reduced gait speeds, impaired balance and dependency, among other causes [6]. It is well known that PwD have a higher risk of falls, and therefore more hip fractures, and often have poor clinical outcomes. In particular, frail PwD are more likely to suffer a further hip fracture within three years [7]. Deficiency in mobility and basic activities of daily living are common through recovery from hip fractures in this vulnerable group. In fact, in Western countries, around 10–20% of patients with hip fractures end up institutionalized within 6–12 months [8].

### Background

When PwD are admitted to hospital after a hip fracture, the busy, unfamiliar setting of a high-tech hospital’s acute unit can be challenging, causing anxiety and distress in patients. They face the discomfort of being in a strange place and there is often a lack of a clear vision on how to care for them as priorities are usually focused on the reason for admission in acute care or physical care needs [9]. Thus, providing care for hospitalized PwD is also challenging for healthcare professionals due to the lack of dementia-friendly care pathways, environments and education, as well as staffing levels [10]. For instance, pain management can be difficult because PwD have difficulties expressing their level of pain [11]. Although many valid instruments have been developed to assess pain in dementia, adequate pain management is still not properly implemented [12]. Nurses find it hard to differentiate pain from behavioral disturbances. Barriers include a lack of multidisciplinary communication or workload pressure [13]. According to the OECD, Spain has 5.9 nurses per 1000 inhabitants [14], and the mean staffing ratio of patients to nurses is 12.7, much higher than other European countries. An increase in nurse workload is associated with a higher patient death rate [15] and missed care [16].

Dementia is associated with malnutrition [17], and other comorbidities are commonly present as well. Reduced intake is often due to pain, poor mobility, being confined to bed and anorexia of aging, which causes a loss of muscle mass and increases the probability of fractures. Malnutrition has a negative impact on functional recovery and mortality in patients with hip fractures [18]. Thus, comorbidities, malnutrition and immobility make them prone to develop pressure ulcers, not only in the hospital setting but also in the community and long-term care [19]. Furthermore, other post-operative complications, such as wounds or urinary tract or respiratory infections, are likely to develop in PwD. These complications also result in prolonged lengths of stay, readmissions, higher costs and reduced physical and social capacities [20]. In fact, after having surgery, one-third of these patients die within a year, and this rate increases with time [21].

There is a lack of specific protocols for dementia care in the acute hospitalization setting, as well as dementia-friendly units. They are admitted to wards due to an acute condition and general guidelines are followed. New strategies need to be considered for patient-centered care in dementia, especially in acute hospitalization, where patients are out of their familiar surroundings. Focusing on patient-centered care could improve outcomes in both patients and healthcare workers. In order to develop new strategies, it is key to know these patients’ overall profile, their outcomes and how they progress after the surgical procedure.

The aim of this study was to analyze the characteristics of patients with dementia and cognitive impairment hospitalized with a proximal femur fracture requiring surgery.

## 2. Materials and Methods

This is a multicentric descriptive longitudinal study.

### 2.1. Setting

The study was conducted in three traumatology units representing high-technology public hospitals across Spain: Hospital Clinic, Barcelona (Catalonia), Hospital Universitario Marqués de Valdecilla (Cantabria) and Hospital Universitario de Navarra (Navarra).

### 2.2. Participants

Participants were people with dementia or cognitive impairment (*n* = 174) hospitalized with a proximal femur fracture requiring surgery. Consecutive recruitment was conducted as they were admitted.

Inclusion criteria were the following:

- Patients aged 65 or older hospitalized for surgery.
- A score of 5 or less in the Short Portable Mental Status Questionnaire (SPMSQ) test [22,23]. Moreover, records on dementia diagnosis or physician assessment from the Emergency Department were consulted. Information was validated by the proxy.
- Living with an informal caregiver or receiving a caregiver visit at least 3 times per week.
- Signed informed consent form provided by the patient or by the patient’s legal representative.

Exclusion criteria were as follows:

- Patients younger than 65.
- Psychiatric symptoms or Korsakoff’s syndrome.
- No informed consent form.
- No IC.

### 2.3. Data Collection

Data collection took place between August 2018 and December 2019 at the following time points following PwD admission to hospital: Within 24 hours of ward admission, on discharge, one month after discharge in the outpatient traumatology visit and at a three-month follow-up (phone call). Data were collected by trained nurses with extensive experience in geriatric orthopedics, and all received training on the study protocol and data-collection procedures for patients and their caregivers. Data were collected after study approval was received from all the participating Hospitals’ Ethics Committees and signed informed consent was provided by study subjects.

### 2.4. Measures

Measurements collected in PwD included sociodemographic and clinical data (the use of physical restraints, the number of falls and pressure ulcers).

**Figure 1:**
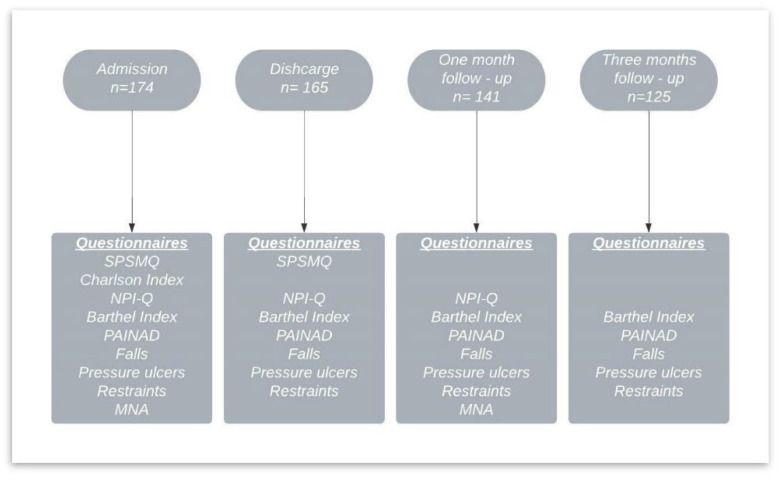
Data collection and measurements.

Functional status was measured with the Barthel Index [24,25]; comorbidities were measured with the Charlson Comorbidity Index [26,27]; pain was measured with the Pain Assessment in Advanced Dementia Scale (PAINAD) [28,29]; neuropsychiatric symptomatology was evaluated with the Neuropsychiatric Inventory Questionnaire (NPI-Q) [30,31]; and nutritional status was checked using the Mini Nutritional Assessment (MNA) [32]. All questionnaires used were the Spanish versions and are valid and reliable. See Figure 1 for data collection and measurements.

### 2.5. Data Analysis

Descriptive data are presented as means and standard deviations (SD) for continuous variables and numbers and percentages (%) for categorical variables. Estimated changes in PwD outcomes were studied with a paired *t*-test for continuous variables and McNemar’s test for categorical variables. Outcomes assessed longitudinal changes from all the collection phases: Baseline (admission), discharge, one-month and three-month follow-up. Confidence intervals of 95% were calculated. All significance tests were two-tailed, and values of *p* < 0.05 were considered significant. All analyses were conducted using the R version 4.1.0. for Windows statistical software package. 

### 2.6. Validity and Reliability/Rigor

This study was conducted following the STROBE reporting standard for cohort studies [33]. Three hospitals were selected to amplify the sample size and increase its representativeness of Spain.

## 3. Results

### 3.1. Sample Characteristics at Baseline

Participants in this study (*n* = 174) were mainly women (81.6%), and the mean age was 90.7 ± 6.3 years old. Of these, most had a diagnosis of dementia (*n* = 120), and the average time since diagnosis was 5.8 ± 4.3 years. The type of dementia was mostly unknown (39.7%) and 30.5% had a diagnosis of Alzheimer’s disease. According to the Spanish Law of dependency, 42% of participants were independent, 21.8% had recognized severe dependency and 27.6% were greatly dependent.

Participants had a comorbidity Index of 2.3 ± 1.5, measured with the Charlson Index. One-third had at least one comorbidity, one-third had two and another third had more than two comorbidities, mostly circulatory (73%), endocrine-metabolic (36.2%) and nephrological (26.4%).

Behavioral disturbance severity results (*n* = 174), measured with the NPI-Q, were 5.2 ± 5.3 and distress results were 4.5 ± 6.8. These represent low severity and distress in behavioral disturbances. Results are shown in Table 1.

### 3.2. Longitudinal Results

A comparison from baseline to the three-month follow-up was conducted on 125 patients. From patients at baseline (*n* = 174), 31 died during the study (17.8%). Eighteen patients did not complete the last follow-up after three months as they were not willing to continue to participate. Almost half of the patients (44%) were discharged home and the other half went to a nursing home (30.4%) or long-term care facility (25.4%).

Functional state decreased significantly (*p* < 0.001) following Barthel Index measurements (*n* = 122). On admission, 9% of patients were totally dependent, which increased to 38.5% on discharge, and 30.3% remained dependent after 3 months. Despite a slight recovery in the third month, they did not return to the same functional state seen at baseline. In addition, fully independent patients at baseline (4.1%) were no longer independent after three months (0.8%). The percentage of participants with mild dependence decreased significantly (from 43.4% at baseline to 21.3% three months after discharge), shifting to a higher level of dependency.

Falls increased from admission to discharge (88% to 96%) and then plummeted to 7.2% after three months (*p* < 0.001). Falls were registered within the last 30 days. Physical restraints applied on admission were mainly bilateral bedrails (18.8%) and “chair with table” (8.7%). During hospitalization, the use of physical restraints increased. On discharge, 60.7% had bilateral bedrails on and 26.5% were using a belt in the chair/bed. After discharge, bilateral bedrail use remained constant. Mostly all patients remained with the room door open.

Pain on admission was measured with the PAINAD tool, reaching a total score of 2.2 ± 2.39, where 69.2% of participants had mild pain, 25.2% moderate pain and 5.6% intense pain on admission. Upon discharge, 22.4% of PwD still had moderate pain and 4.7% had intense pain. After the three-month follow-up, 3.7% of PwD continued to experience intense pain and 12.1% experienced moderate pain (Table 2).

The number of pressure ulcers, with the majority of them being stage II and III, increased significantly (*p* < 0.001 after one month). Upon admission, 2.4% of PwD had a pressure ulcer, compared with 9.6% on discharge. After one month, the presence of an ulcer increased to 15.2% of patients and decreased to 12.8% three months later. The location of pressure ulcers one month after discharge was mainly on the heels (9.6%) and sacrum/back (3.2%). These results are shown in Table 2. A Supplementary File has been added for a detailed ratio of pressure ulcers to hospitals and nursing.

Nutritional status was measured with MNA at baseline and one-month follow-up (n = 141). The overall MNA score was 17.43 ± 4.4. on admission and decreased to 15.36 ± 4.9 (*p* < 0.001). Results are shown in Table 3.

## 4. Discussion

The overall condition of older people with dementia or cognitive impairment deteriorated after hospitalization and surgery for a hip fracture, especially due to malnutrition and the development of PUs, falls, functional impairment and the use of physical restraints and pain management challenges.

From baseline, participants were already significantly malnourished or at risk of malnutrition. A systematic review found a relationship between frailty and malnutrition in the community setting; older malnourished people were likely to be frail, although only a few frail older people were malnourished. Of those found to be malnourished, 68% were also physically frail, and 25.8% were prefrail [34]. Although we did not measure frailty in our study, our sample had a similar profile, and the results were comparable. We should also consider sarcopenia as an added factor to frailty, where muscle mass, strength and function deteriorate [35]. Our results are similar to those of studies in patients with cognitive impairment, where 30% of patients were malnourished, 56% were at risk and 14% had a normal nutritional status [35]. Chye et al. [36] also reported a lower prevalence of malnutrition in frail older people with cognitive impairment (23% malnourished, 49.2% at risk of malnutrition and 27.7% with normal nutrition). However, we have to consider the median age of participants (66.4 ± 7.8 years) compared to our cohort (90.7 ± 6.3) [36]. Moreover, our study results show statistically significant worsening of nutritional status one month after discharge, a 17.7% rise in malnourished participants from baseline and a reduction in participants with normal nutrition status from 19.1% to 17.7% (*p* <0.001).

Due to malnourishment and frailty, participants were prone to developing pressure ulcers. Another important factor to consider regarding hospitalization after surgery among elderly patients is the development of pressure ulcers. Galivanche et al. [37] reported that 5.15% of patients undergoing hip fracture surgery developed PUs. Our population had a higher rate, as it was rather frail and malnourished and also showed dependency in activities of daily living and pre-existing PUs. This could predict a higher risk of PUs. The three hospitals protected heels with foam dressings as per protocol in all elderly patients at risk of developing PUs and also maintained regular comfort measures, such as the use of pillows and frequent repositioning to avoid pressure. However, heels remained the prime site for PUs. After discharge, the number of PUs kept rising, and those that existed progressed to a more-severe stage. Half of our patients were discharged into long-term care facilities or nursing homes where patients are looked after by care assistants with higher patient ratios and heavier workloads. This could explain some worsening aspects in these patients after discharge.

Regarding the use of physical restraints, there are discrepancies with respect to experiences and beliefs, especially regarding their use on elderly, cognitively impaired patients [38]. In cases of agitation, belts or restraints are used only with medical prescriptions. Our results show physical restraints are still used during hospitalization, especially bilateral bedrails and chairs with tables and belts. Although there was a slight reduction after discharge, their use continued. The RightTimePlaceCare (RTPC) study reported the use of physical restraints in 17.8% of PwD at home and 83.2% in institutional care in Spain, rates that are twice as high as the overall figure for eight other European countries [39].

The functional state of these patients also deteriorated from admission to discharge and the one-month follow-up visit. Total dependency, in most cases, implies the inability to move and thus falls are avoided. According to our results, mobility after surgery could be reduced as patients did not fully recover their functional state. These results are supported by Dyer et al. [8], who found that patients with a high-dependence pre-fracture are less likely to recover their level of independence in activities of daily living. Moreover, Bower et al. [40] indicated that the fear of falling is high among these patients (61% 4 weeks post-fracture and 47% 12 weeks after) and therefore these PwD often cut back on activities and exercise routines, thus worsening their functional state. Balance impairment and mobility limitations are intrinsic factors of falls [41]. In fact, participants had a higher rate of falls one month after their discharge from hospital.

Furthermore, Cunningham et al. [42] indicated that, in the elderly, more physical activity predicts higher functional status as well as a reduction in the risk of fractures. This suggests that falls could probably be prevented if physical activity was adequate in PwD. In addition, in order to enhance mobility, proper pain assessment with recommended scales should be used in PwD or those with cognitive impairment [43].

We used the PAINAD scale for pain assessment, a behavioral observational tool for PwD who are unable to communicate, which focuses on breathing, vocalization, facial expression, body language and consolability [28]. This tool showed a correlation with pain biomarkers in saliva, which confirms its usefulness for assessing pain in PwD [44]. Based on the PAINAD scale results, a significant number of participants remained in pain throughout the study. These results are supported by those of other studies showing that PwD still remain undertreated for pain [45]. Moreover, Nowak et al. [46] found low Barthel scores among institutionalized patients with cognitive impairment inappropriately treated for pain. Our cohort received scheduled paracetamol and metamizole during hospitalization, and PRN (as needed) analgesia at home. Implementing standardized protocols to guide nurses and ICs in decision-making is essential to ensure better control of pain in PwD [47]. It should be pointed out that if patients’ pain is under control, they would probably move more and consequently improve their overall physical condition.

Our results indicate that the overall condition of PwD deteriorated following surgical hip replacement. PwD did not recover their initial functional capacity, and pressure ulcers and malnutrition also increased. These complications seem to delay patient recovery and wellness. Therefore, the healthcare system should implement new patient-centered care strategies to improve PwD outcomes and wellbeing, especially in the acute setting. This increasing population group needs close follow-ups to improve these indicators and to view the process from a holistic perspective. For instance, dementia-friendly wards should be considered to care for all PwD hospitalized instead of admitting them into diagnosis-related wards. Specialized staff could have a specific care path for PwD and also consider their caregivers during their hospitalization.

This study has some limitations. While data from patients during hospitalization and outpatient visits at one-month post-discharge were collected through interviews by nurse researchers, the three-month follow-up was conducted by phone. Telephone interviews had lower response rates; some informal caregivers declined to complete them, probably because the questionnaires were too long, and they already had a large burden. Although data were collected by different interviewers, they all followed the same protocol.

A strength of this study is providing an overall profile of this vulnerable group hospitalized in three high-technology hospitals representing different areas in Spain. Many studies have been conducted in the community and long-term or residential setting, but not in acute hospitalization where units are not dementia-friendly. Further research would be useful to gain a deep understanding of each item evaluated in this study.

## 5. Conclusions

Hospitalization for elderly PwD undergoing surgical procedures due to hip fractures negatively impacted their overall status. During the study timeframe, our cohort became more dependent and malnourished; the number of pressure ulcers increased; physical restraints were used more often; and pain was not properly controlled. Conversely, the number of falls and related injuries decreased significantly after three months. It is necessary to implement effective strategies to improve overall outcomes of PwD requiring hospitalization.

## Figures and Tables

**Table 1:** Characteristics of patients at admission.

Patient Characteristics	Total (*n* = 174)
	Mean ± SD
Age, years	90.7 ± 6.3
Gender, female	*n* (%)
Patients with dementia	142 (81.6)
Type of dementia	*n* (%)
Alzheimer’s	53 (30.5)
Unknown	69 (39.7)
Vascular dementia	26 (14.9)
Other	26 (14.9)
Time since diagnosis, years (*n* = 120)	5.8 ± 4.3
Degree of care dependency	*n* (%)
0	73 (42.0)
1	15 (8.6)
2	38 (21.8)
3	48 (27.6)
PwD diseases	*n* (%)
Neurological	137 (78.7)
Circulatory	127 (73.0)
Endocrine-metabolic	63 (36.2)
Respiratory	21 (12.1)
Nephrological	46 (26.4)
Oncological	20 (11.5)

Data are presented as number (percentage) or means ± standard deviation.

**Table 2:** Estimated change in outcomes during all study phases (hospital admission, discharge, 1-month follow-up and 3-month follow-up) (*n* = 125).

Variable	Admission	Discharge	One Month Follow-Up	Three Months Follow-Up	*p*-Value †	*p*-Value ‡	*p*-Value §
Functional state (Barthel) (n = 122)	56.05 ± 26.86	27.98 ± 20.46	32.55 ± 24.27	35.16 ± 25.45	<0.001	<0.001	<0.001
Total dependence (<20)	11 (9.0)	47 (38.5)	43 (35.2)	37 (30.3)			
Severe (20–35)	23 (18.9)	38 (31.1)	32 (26.2)	35 (28.7)			
Moderate (40–55)	30 (24.6)	26 (21.3)	28 (23.0)	23 (18.9)			
Mild dependence (60–95)	53 (43.4)	11 (9.0)	18 (14.8)	26 (21.3)			
Independence (100)	5 (4.1)	0 (0)	1 (0.8)	1 (0.8)			
Physical restraints (*n* = 117)							
Belt in chair/Belt in bed	7 (6.0)	31 (26.5)	21 (17.9)	15 (12.8)	<0.001	0.001	0.061
Chair with table	10 (8.7)	14 (12.2)	20 (17.4)	26 (22.6)	0.480	0.066	0.008
Bilateral bedrails	22 (18.8)	71 (60.7)	49 (41.9)	42 (35.9)	<0.001	<0.001	0.004
Pain (PAINAD) (*n* = 110)	2.2 ± 2.39	1.69 ± 2.27	1.22 ± 2.13	1.23 ± 2.14	0.021	<0.001	0.002
Mild (0–3)	74 (69.2)	78 (72.9)	88 (82.2)	90 (84.1)			
Moderate (4–6)	27 (25.2)	24 (22.4)	15 (14.0)	13 (12.1)			
Intense (7–10)	6 (5.6)	5 (4.7)	4 (3.7)	4 (3.7)			
Pressure ulcers							
Presence	3 (2.4)	12 (9.6)	19 (15.2)	16 (12.8)	0.008	<0.001	0.004
Stage 2	2 (1.6)	10 (8.0)	10 (8.0)	9 (7.2)			
Stage 3	1 (0.8)	2 (1.6)	7 (5.6)	6 (4.8)			
Stage 4	0 (0)	0 (0)	2 (1.6)	1 (0.8)			
Location of pressure ulcer							
Sacrum/Back	2 (1.6)	5 (4.0)	4 (3.2)	3 (2.4)			
Heel	1 (0.8)	7 (5.6)	12 (9.6)	12 (9.6)			
Falls (presence)	110 (88)	120 (96)	24 (19.2)	9 (7.2)	0.004	<0.001	<0.001
Falls (frequency)	1.14 ± 1.01	1.22 ± 1.16	0.26 ± 0.65	0.13 ± 0.52	0.086	<0.001	<0.001
0	15 (12.0)	5 (4.0)	101 (80.8)	116 (92.8)			
1	94 (75.2)	108 (86.4)	19 (15.2)	5 (4.0)			
2	9 (7.2)	5 (4.0)	4 (3.2)	1 (0.8)			

Data are presented as number (percentage) or means ± standard deviation. † Comparison between admission and discharge. ‡ Comparison between admission and one-month follow-up. § Comparison between admission and three-month follow-up.

**Table 3:** Comparison of nutritional status between admission and one-month follow-up (*n* = 141).

Variable	Admission	One Month Follow-Up	*p* Value
**Nutritional status (MNA)**	17.43 ± 4.4	15.36 ± 4.9	<0.001
Malnutrition (<17)	51 (36.2)	76 (53.9)	
Risk of malnutrition (17–23.5)	63 (44.7)	40 (28.4)	
Normal (24–30)	27 (19.1)	25 (17.7)	

Data are presented as number (percentage) or means ± standard deviation.

## Data Availability

Data presented in this study are available on request from the corresponding author; data are not publicly available.

## Supplementary Materials

The following are available online at https://www.mdpi.com/article/10.3390/ijerph19052799/s1, Supplementary File: Detailed Ratio of Pressure Ulcers to Hospitals and Nursing.
